# Supplementary material for: Tuning Plasmonic Properties of Gold Nanoparticles by Employing Nanoscale DNA Hydrogel Scaffolds
Source: Biosensors (Basel). 2022 Dec 24;13(1):20. doi: 10.3390/bios13010020 (PMC9855890; doi:10.3390/bios13010020)
Supplement: Supplementary file 1 [file biosensors-13-00020-s001.zip › biosensors-2086275-supplementary.pdf]

biosensors

MDPI

Supporting Information

Tuning Plasmonic Properties of Gold Nanoparticles by Employing Nanoscale DNA Hydrogel Scaffolds

Mohzibudin Quazi, Taeyoung Kim, Jinhwan Yang and Nokyoung Park *

Department of Chemistry and The Natural Science Research Institute, 116 Myongji-ro, Yongin, Gyeonggi-do, Republic of Korea
* Correspondence: pospnk@mju.ac.kr

Table S1. Specification of varied X-DNAs and their sequences.

(1) 36 bp XDNA sequences.

Strand label	Sticky Ends	Main body segment 5'—3'
X-01		CGA CCG ATG AAT AGC GGT CAG ATC CGT ACC TAC TCG
X-02	GATC	CGA GTA GGT ACG GAT CTG CGT ATT GCG AAC GAC TCG
X-03	GATC	CGA GTC GTT CGC AAT ACG GCT GTA CGT ATG GTC TCG
X-04	GATC	CGA GAC CAT ACG TAC AGC ACC GCT ATT CAT CGG TCG

(2) 56 bp XDNA sequences.

Strand label	Sticky Ends	Main body segment 5'—3'
X-01		5Phosp/AGC GGT CCA CTG GAT CCG CAT GAG GTA GGA CGA CAT TCG CCG TAA GCA CAC AGA TC
X-02	ACGT	5Phosp/ACG TGA TCT GTG TGC TTA CGG CGA ATG TCG TCA CAG CAC CGA ATC AGC CTG CTG CGT ATT
X-03	ACGT	5Phosp/ACG TAA TAC GCA GCA GGC TGA TTC GGT GCT GTC TGA CTT CAG CTC CAT GAG TAC GCT GTA
X-04	ACGT	5Phosp/ACG TTA CAG CGT ACT CAT GGA GCT GAA GTC AGC TAC CTC ATG CGG ATC CAG TGG ACC GCT

(3) 76 bp XDNA sequence.

Strand label	Sticky Ends	Main body segment 5'—3'
X-01		ACC GAT GAA TAG CGG TCC ACT GGA TCC GCA TGA GGT AGG ACG ACA TTC GCC GTA AGC ACA CAG ATC CGT ACC TAC T
X-02	ACGT	5Phosp/ ACG TAG TAG GTA CGG ATC TGT GTG CTT ACG GCG AAT GTC GTC ACA GCA CCG AAT CAG CCT GCT GCG TAT TGC GAA CGA CT
X-03	ACGT	5Phosp/ ACG TAG TCG TTC GCA ATA CGC AGC AGG CTG ATT CGG TGC TGT CTG ACT TCA GCT CCA TGA GTA CGC TGT ACG TAT GGT CT
X-04	ACGT	5Phosp/ ACG TAG ACC ATA CGT ACA GCG TAC TCA TGG AGC TGA AGT CAG CTA CCT CAT GCG GAT CCA GTG GAC CGC TAT TCA TCG GT

Table S2. Dgel Synthesis flow table.

Concentration / Base pair (bp)	[150 µM]	[100 µM]	[50 µM]	[25 µM]
36bp X-DNA	Dgel (36bp X-DNA[150 µM])	Dgel (36bp X-DNA[100 µM])	Dgel (36bp X-DNA[50 µM])	Dgel (36bp X-DNA[25 µM])
56bp X-DNA	Dgel (56bp X-DNA[150 µM])	Dgel (56bp X-DNA[100 µM])	Dgel (56bp X-DNA[50 µM])	Dgel (56bp X-DNA[25 µM])
76bp X-DNA	Dgel (76bp X-DNA[150 µM])	Dgel (76bp X-DNA[100 µM])	Dgel (76bp X-DNA[50 µM])	Dgel (76bp X-DNA[25 µM])

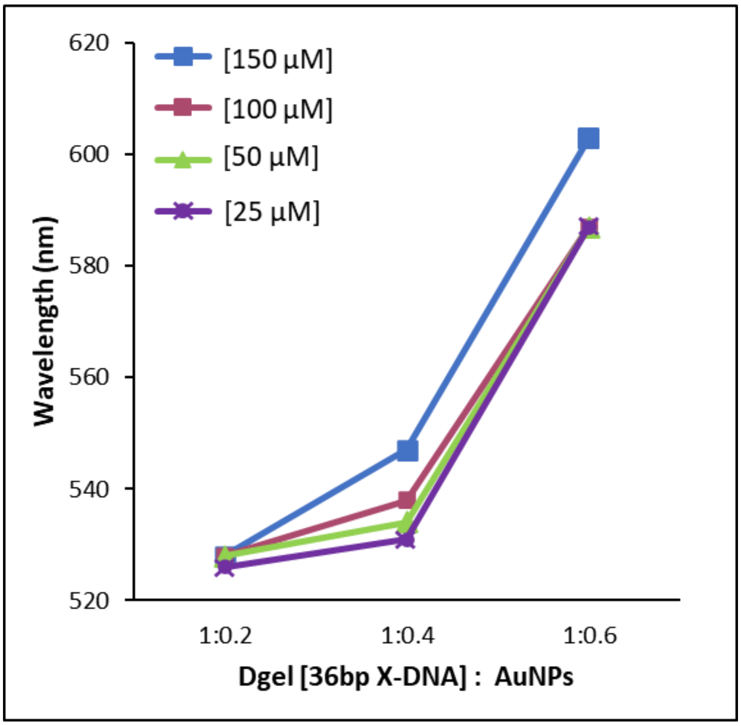

Figure S1. Plasmonic absorbance peaks of 36 bp XDNA's Dgel: AuNPs nano assemblies at XDNA's variable molar concentration and with different molar ratios of Dgel [36bp X-DNA]: AuNPs.

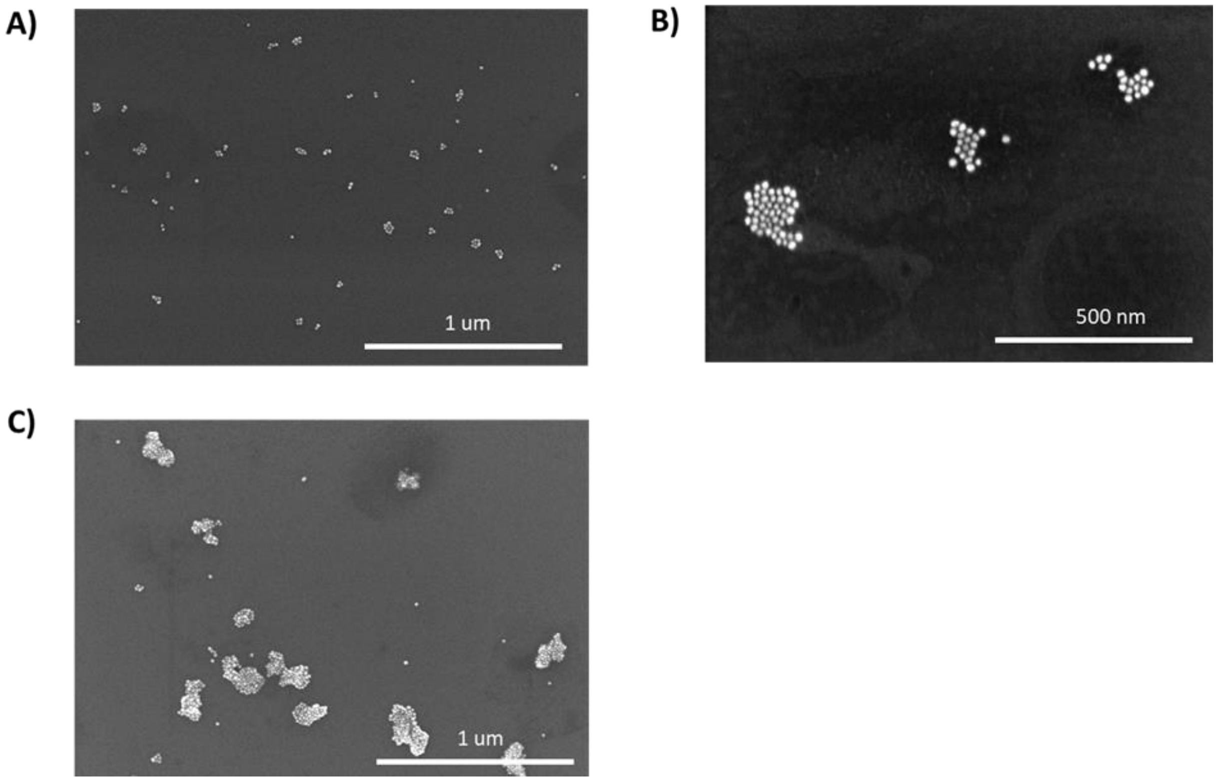

Figure S2. SEM image analysis of (36bp X-DNA [150uM] Dgel): AuNPs nano assemblies at variable molar ratios. A) Dgel: AuNPs 1: 0.2 B) Dgel: AuNPs 1: 0.4 C) Dgel: AuNPs 1: 0.6.

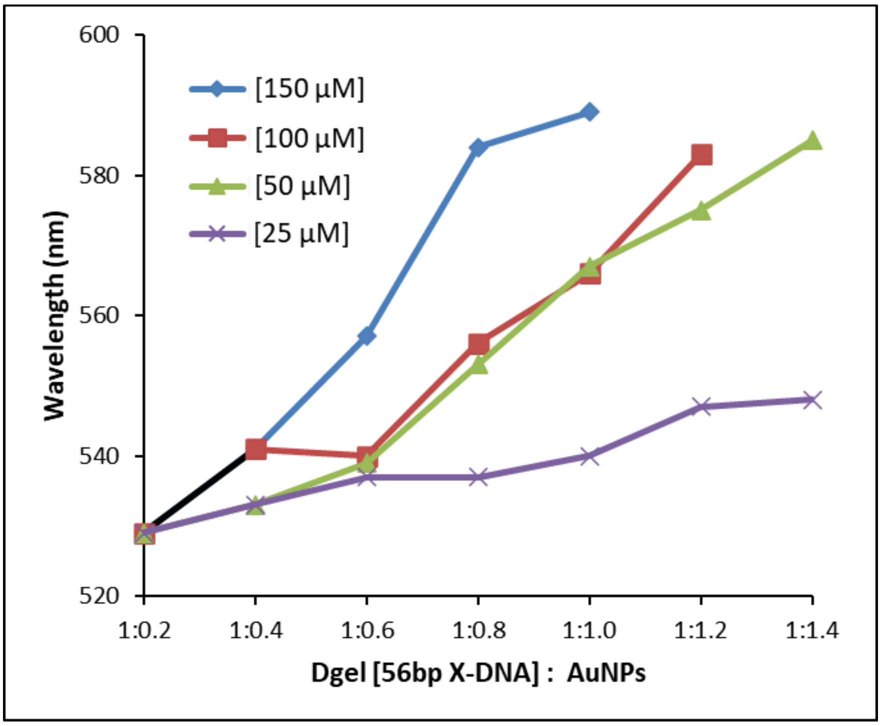

Figure S3. Plasmonic absorbance peaks of 56 bp XDNA's Dgel: AuNPs nano assemblies at XDNA's variable molar concentration and with different molar ratios of Dgel [56bp X-DNA]: AuNPs.

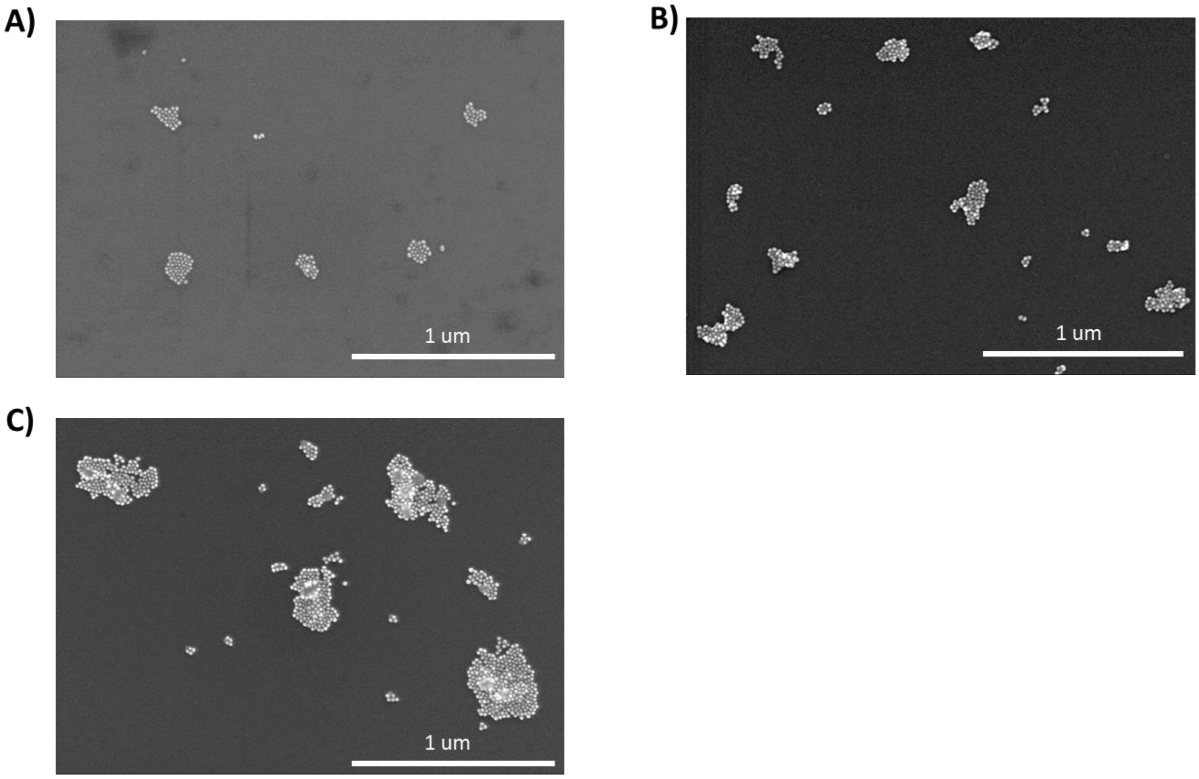

Figure S4. SEM image analysis of (56bp X-DNA [150uM] Dgel): AuNPs nano assemblies at variable molar ratios. A) Dgel: AuNPs 1: 0.2 B) Dgel: AuNPs 1: 0.4 C) Dgel: AuNPs 1: 0.6.

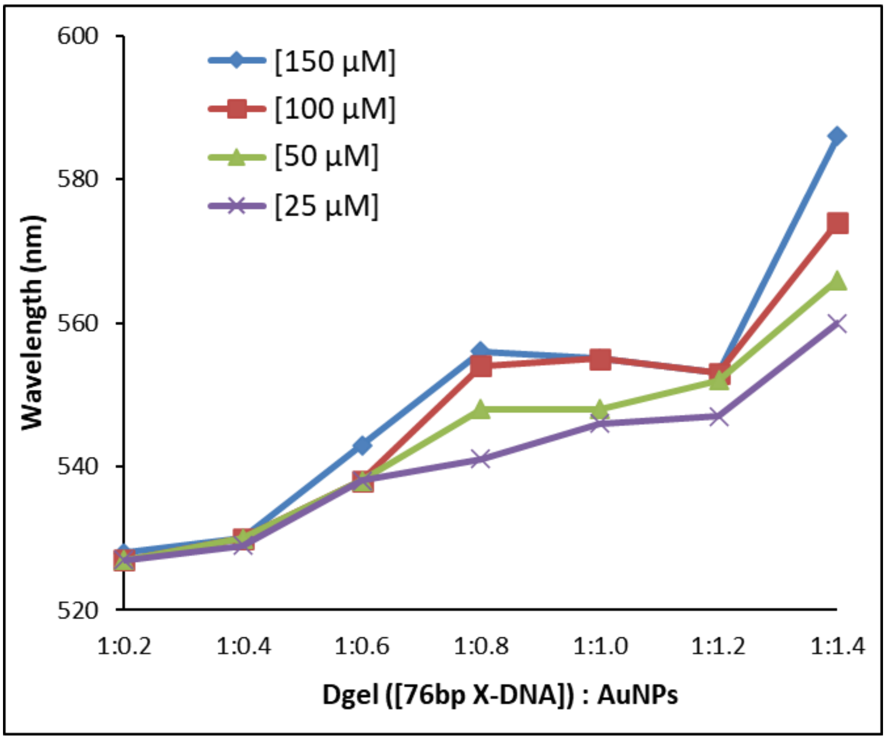

Figure S5. Plasmonic absorbance peaks of 76 bp XDNA's Dgel: AuNPs nano assemblies at XDNA's variable molar concentration and with different molar ratios of Dgel [76bp X-DNA]: AuNPs.

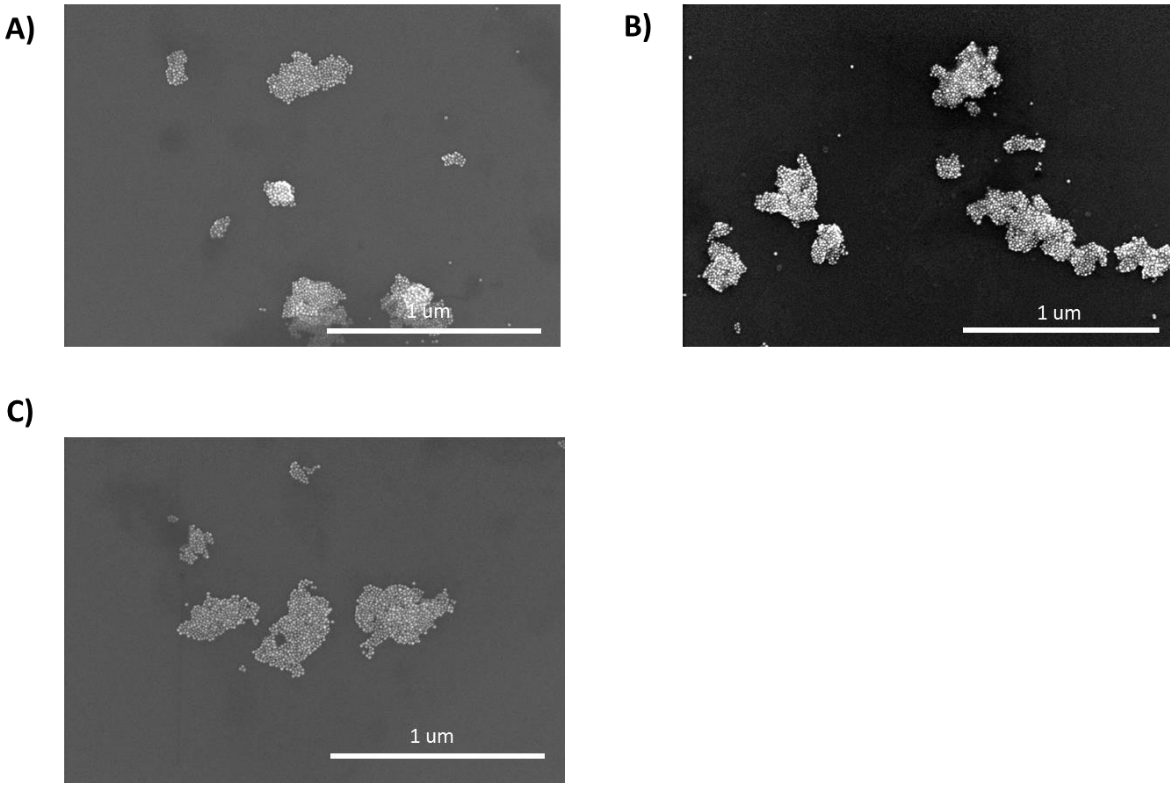

Figure S6. SEM image analysis of (56bp X-DNA [150uM] Dgel): AuNPs nano assemblies at variable molar ratios. A) Dgel: AuNPs 1: 0.4 B) Dgel: AuNPs 1: 0.8 C) Dgel: AuNPs 1: 1.2.

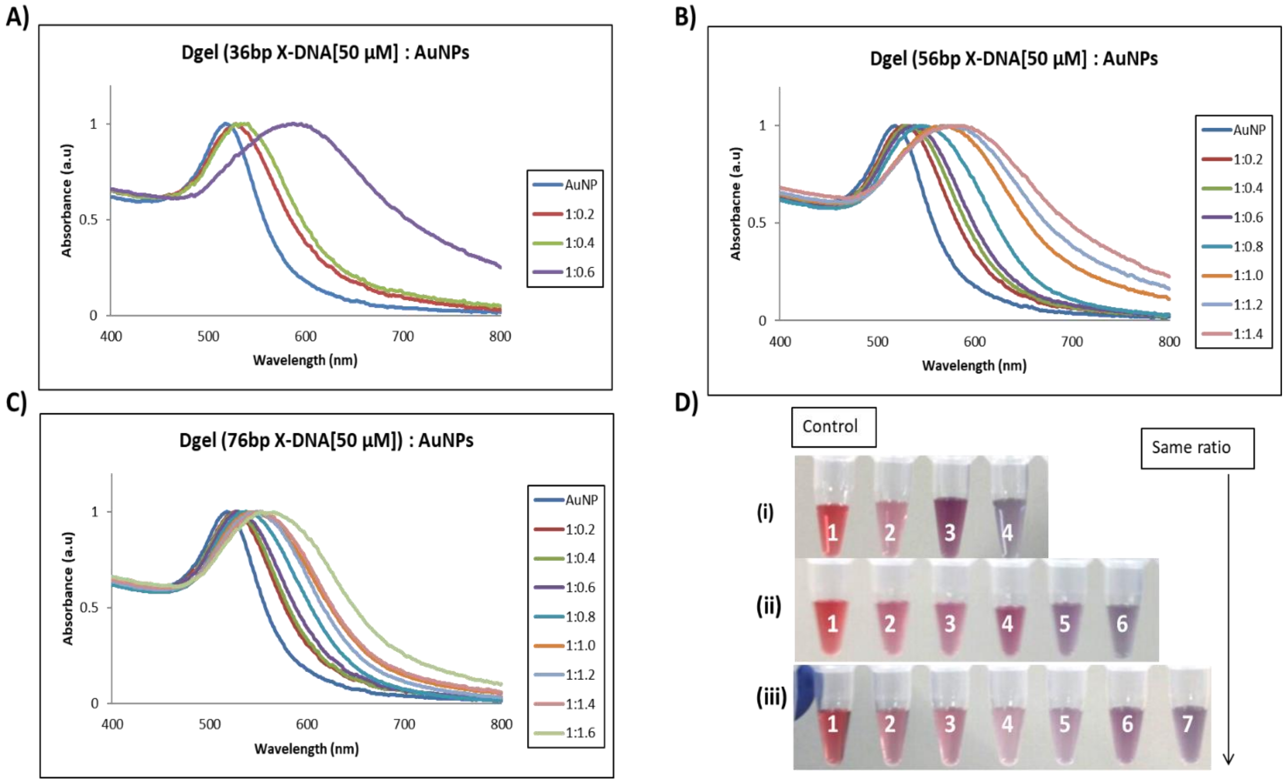

Figure S7. Surface plasmonic shift observations in Dgel: AuNPs nano assemblies by UV visible and digital images. A), B) and C) Uv visible studies of Dgel-AuNPs assembly and change in SPR properties. The concentrations of X-DNA-engineered Dgels were constant whereas the AuNPs

ratios were varied to study the Dgel-AuNPs assembly and redshift. D) The obtained digital images after the nano assemblies at varied ratios of different X-DNA engineered Dgels.

[P.S. - The graphs are identical to the graph presented in the main figures of the manuscript. Here, added to correlate with obtained digital images of AuNPs.]References

1. Dreaden, E. C., Alkilany, A. M., Huang, X., Murphy, C. J., & El-Sayed, M. A. (2012). The golden age: gold nanoparticles for biomedicine. Chem. Soc. Rev., 41(7), 2740–2779. https://doi.org/10.1039/C1CS15237H
2. Quazi, M. Z., Lee, U., Park, S., Shin, S., Sim, E., Son, H., & Park, N. (2021). Cancer Cell-Specific Enhanced Raman Imaging and Photothermal Therapeutic Effect Based on Reversibly pH-Responsive Gold Nanoparticles. ACS Applied Bio Materials, 4(12), 8377–8385. https://doi.org/10.1021/acsabm.1c00946
3. Song, J., Hwang, S., Im, K., Hur, J., Nam, J., Hwang, S., Ahn, G. O., Kim, S., & Park, N. (2015). Light-responsible DNA hydrogel-gold nanoparticle assembly for synergistic cancer therapy. Journal of Materials Chemistry B, 3(8), 1537–1543. https://doi.org/10.1039/c4tb01519c
